# Effect of mechanical ventilation versus spontaneous breathing on abdominal edema and inflammation in ARDS: an experimental porcine model

**DOI:** 10.1186/s12890-020-1138-6

**Published:** 2020-04-25

**Authors:** Silvia Marchesi, Göran Hedenstierna, Aki Hata, Ricardo Feinstein, Anders Larsson, Anders Olof Larsson, Miklós Lipcsey

**Affiliations:** 1 Hedenstierna Laboratory, Department of Surgical Sciences, Uppsala University, 75185 Uppsala, Sweden; 2 Department of Medical Sciences, Clinical Physiology, Uppsala University, Uppsala, Sweden; 3 National Veterinary Institute, Uppsala, Sweden; 4 Section of Clinical Chemistry, Department of Medical Sciences, Uppsala University, Uppsala, Sweden

**Keywords:** ARDS, Mechanical ventilation, Spontaneous ventilation, Cytokines, Edema, Abdominal inflammation

## Abstract

**Background:**

Mechanical ventilation (MV), compared to spontaneous breathing (SB), has been found to increase abdominal edema and inflammation in experimental sepsis. Our hypothesis was that in primary acute respiratory distress syndrome (ARDS) MV would enhance inflammation and edema in the abdomen.

**Methods:**

Thirteen piglets were randomized into two groups (SB and MV) after the induction of ARDS by lung lavage and 1 h of injurious ventilation.

1. SB: continuous positive airway pressure 15 cmH_2_O, fraction of inspired oxygen (FIO_2_) 0.5 and respiratory rate (RR) maintained at about 40 cycles min^− 1^ by titrating remifentanil infusion.

2. MV: volume control, tidal volume 6 ml kg^− 1^, positive end-expiratory pressure 15 cmH_2_O, RR 40 cycles min^− 1^, FIO_2_ 0.5.

Main outcomes: abdominal edema, assessed by tissues histopathology and wet-dry weight; abdominal inflammation, assessed by cytokine concentration in tissues, blood and ascites, and tissue histopathology.

**Results:**

The groups did not show significant differences in hemodynamic or respiratory parameters. Moreover, edema and inflammation in the abdominal organs were similar. However, blood IL6 increased in the MV group in all vascular beds (*p* < 0.001). In addition, TNFα ratio in blood increased through the lungs in MV group (+ 26% ± 3) but decreased in the SB group (− 17% ± 3).

**Conclusions:**

There were no differences between the MV and SB group for abdominal edema or inflammation. However, the systemic increase in IL6 and the TNFα increase through the lungs suggest that MV, in this model, was harmful to the lungs.

## Background

Mechanical ventilation (MV) is a cornerstone in the treatment of respiratory failure (e.g., acute respiratory distress syndrome, ARDS). Although MV is commonly associated with lung injury [1] and ventilator-induced lung injury (VILI) is one of the most studied topics in respiratory intensive care research [2, 3]. Additional to its effects on the lungs, MV can potentially impact other areas of the body (e.g., the abdomen). However, the effect of MV on the abdomen has not been extensively studied.

The positive pressure produced in the thorax during MV is transmitted to the abdominal compartment. An increase in intra-abdominal pressure (IAP) affects lungs [4, 5] producing atelectasis [6], impaired function [7] and, indirectly, inflammation and edema [8]. On the other side, an increase in thoracic pressure due to MV [9] may have an effect on inflammation and edema in the abdominal compartment. In fact, the decrease in venous return related to high positive end-expiratory pressure (PEEP) ventilation [10] has been well described. In addition, a reduction on portal flow due to a vasoconstrictive reaction related directly to PEEP [11] has also been described. Consequently, MV can affect hemodynamics both systemically and locally (e.g., by enhancing hepatic production of inflammation mediators [12]).

Increased abdominal edema and inflammation as a result of MV has been demonstrated in previous studies. Abdominal edema and decreased lymphatic drainage [13] were associated with mechanical ventilation and worsened by a high level of PEEP, whereas abdominal inflammation was related to MV irrespective of PEEP levels [14, 15].

In these studies an endotoxemic model was used [13, 14]. The hallmark of this model is a systemic inflammatory response with a substantial increase in inflammatory markers and the development of edema. Thus, these studies report the combined effect of endotoxemia and MV on abdominal edema (ascites) and inflammation. However, available data are confounded by differences in respiratory mechanics. The most important differences found in earlier research are related to a different respiratory rate (not controlled in spontaneous breathing [SB] animals) and in PEEP level application. Hence, the effect of mechanical ventilation contra SB on abdominal inflammation and edema in a non-septic model has not been investigated.

We hypothesized that MV would induce more abdominal edema and inflammation than SB in an experimental model of ARDS.

This study aimed to compare the abdominal inflammatory response and edema formation in mechanically ventilated and spontaneously breathing piglets. To avoid potential confounding factors, we applied a primary ARDS model, maintaining a similar circulation, respiratory rate and applying the same level of PEEP.

The primary endpoints were edema and inflammation in the abdomen up to 6 h after the induction of ARDS.

## Methods

The study was approved by the Animal Research Ethical Committee of Uppsala University (dnr C 145/14). Thirteen male piglets 2–3 months old with a mean weight of 25.6 ± 1.3 kg were included in the studied. Experiments were performed during day-time in an equipped laboratory. Piglets came from a certified farm and they had free access to food and water till 12 h before the transfer to the laboratory, where experiments were started immediately after pigs’ arrival.

Animals were randomized using a casual number assignment into two groups.

In both groups a mild to moderate ARDS was induced using alveolar lavages and 1 h of injurious ventilation [16].

In one group piglets were mechanically ventilated using a protective ventilation approach (MV group, *n* = 7) for the entire 6-h observation time. In the other group piglets were left to breathe spontaneously (SB group, *n* = 6) after the induction of ARDS.

### Preparation

Animals were pre-medicated with Zoletil Forte (tiletamine and zolazepam) 6 mg kg^− 1^ and Rompun (xylazine) 2.2 mg kg^− 1^ intramuscularly. After 5 to 10 min, the animals were placed in the supine position on an operating table and monitored with an EKG and a SpO_2_ probe. A bolus of fentanyl 10–20 μg kg^− 1^ was given, a tracheotomy was performed and an 8.0 mm ID endotracheal tube was inserted (Mallinckrodt Medical, Ireland).

Ventilation was started in volume-controlled mode by a Servo-I ventilator (Maquet, Sweden) with a tidal volume (V_T_) of 8 mL kg^− 1^, inspiratory:expiratory ratio (I:E) 1:2, fraction of inspired oxygen fraction (FIO_2_) 0.5, respiratory rate (RR) 25 cycles min^− 1^ and PEEP 5 cmH_2_O for the entire preparation time.

Anesthesia was then maintained with a continuous intravenous (i.v.) infusion of ketamine 30 mg kg^− 1^ h^− 1^ and midazolam 0.1 mg kg^− 1^ h^− 1^ in a saline solution and a separate remifentanil in a syringe pump (0.1–0.2 μg kg^− 1^ min^− 1^). After checking that anesthesia was sufficient to prevent responses to painful stimulation, muscle relaxation was added as a continuous i.v. infusion of rocuronium 3 mg kg^− 1^ h^− 1^.

A triple-lumen, thermistor-tipped, balloon catheter (Swan-Ganz catheter, 7 Fr) was placed in the pulmonary artery from the right external jugular vein. Through the same access, a central venous catheter was inserted. A neck artery was cannulated. A second triple-lumen, thermistor-tipped, balloon catheter was placed in the hepatic vein from the left internal jugular vein (positioning was ascertained by fluoroscopy) and a 4 Fr catheter in the portal vein via the splenic vein. For the introduction of the catheter in the splenic vein, a laparotomy was performed to expose the spleen. The vein was cannulated with an 18 G peripheral catheter, a 4 Fr catheter inserted over metal guidewire (Seldinger technique). The spleen was carefully repositioned in the abdomen and the peritoneum, muscle layers and skin were sutured. The catheters were used for blood sampling and pressure measurements. Cardiac output was measured by thermodilution using the thermistor-tipped catheter. A bladder catheter was inserted to collect urine and to measure IAP.

### Protocol

After baseline measurements, the animals were randomized to the two intervention groups (MV, SB). ARDS was then obtained using a double hit method [16]. First, the animals underwent five lung lavages (30 mL kg^− 1^ of warmed isotonic saline) to wash out alveolar surfactant and decrease the PO_2_/FIO_2_. The lavages were followed by 60 min of injurious MV (PEEP 0 cmH_2_O, mean plateau pressures 40 cmH_2_O, RR 20 cycles min^− 1^, FIO_2_ 1.0 and I:E 1:2).

After the induction of ARDS, hemodynamic and respiratory measurements were registered.

In the MV group protective MV (V_T_ 6 mL kg^− 1^, FIO2 0.5, RR 35/40 cycles min^− 1^, I:E 1:2, PEEP 15 cmH_2_O) was performed during the observation period (6 h). Rocuronium infusion was maintained.

In the SB group the pigs were left in SB with continuous positive airway pressure (CPAP) at 15 cmH_2_O for 6 h. At the end of the preparation, rocuronium infusion was discontinued. Remifentanil infusion was titrated to maintain an appropriate analgesia and a respiratory rate similar to the MV group.

After 6 h of observation, the animals were sacrificed using an injection of potassium chloride (KCl: 100 mmoL) through the central venous catheter. During the post-mortem exam, samples from the intestine (duodenum and ileum), liver, spleen and ascites were collected for histopathological analysis, cytokines concentration measurements and wet-dry weight.

### Data presented

#### Hemodynamics and respiratory function data

Hemodynamic measurements were registered at baseline, after the induction of ARDS and every hour during the 6-h observation period.

Respiratory parameters were registered continuously by a data collection system.

At baseline, after ARDS induction and each hour during the observation phase, blood gases from artery and pulmonary artery were sampled for blood analyses (Radiometer 300, Denmark).

#### Inflammation

Inflammation was assessed by measuring cytokines in blood, ascites and tissues (duodenum, liver, spleen). TNFα, IL6 and IL1b were measured using ELISA as described previously [17]. Blood samples were taken from four vascular beds (artery, pulmonary artery, portal vein, hepatic vein) at baseline and before euthanasia, whereas ascites samples and organs were only taken post-mortem.

The TNFα and IL6 increase or decrease passing through lungs and liver were calculated as the percentage difference between arterial and pulmonary artery blood concentrations and between hepatic and portal vein blood concentration and used to assess the production and metabolism of cytokines in lungs and liver. Pulmonary and liver ratios were compared in the two study groups.

In abdominal organs tissue histopathological analysis was performed (in duodenum, ileum, liver, spleen). To analyze data on inflammation the pathologist used an inflammation score [18] that took into account the number and type of leukocytes, the localization of leukocytes and the type, intensity and extension of damage (necrosis, exfoliation, degeneration, apoptosis or erosion).

The biochemistry analyses were performed by persons blinded to the protocol, as were the histopathological analyses (performed by a veterinary pathologist).

#### Edema

Edema was assessed by comparing wet and dry weight of the intestine, liver and spleen.

In addition, a veterinary pathologist, blinded to the protocol, gave an approximated quantitative description of edema in the same organs using a score from 0 to 3 and describing potentially relevant findings.

Hemoglobin concentration in blood was used as a marker of hemoconcentration and capillary leakage.

#### Statistical analysis

Data were assessed for normality. A comparison between the MV and SB group for continuous variables at different time points was performed using a two-way analysis of variance (ANOVA) with post-hoc tests for multiple comparisons. For non-parametric data, the Mann-Whitney test was used. Cytokine data were log-transformed [19] and a comparison between groups was done using two-way ANOVA and multiple comparisons. For correlation tests of normally distributed continuous variables, Pearson’s coefficient was calculated. Data analyses and images were performed using R version 3.6.0 and Prism GraphPad version 8.0.2. Data are presented as mean and ± standard deviation unless otherwise stated. A *p* < 0.05 was considered to indicate statistical significance.

## Results

### ARDS model

As planned, a mild to moderate ARDS was created. PaO_2_/FIO_2_ was 241 ± 85 after the induction of ARDS without any difference between the two groups. Likewise, both RR and V_T_ were the same in the two groups.

The PaO_2_ value increased over time in both groups (*p* = 0.04), but was higher in the SB than in the MV group from the 5th hour onwards. PaCO_2_ decreased over time in both groups (*p* = 0.005) though more rapidly in the SB group (time*group effect p = 0.04). Respiratory variables are reported in Table 1.

**Table 1:** Respiratory parameters reported as Mean ± Standard Deviation in both groups

	SB group							MV group						
	RR	PEEP	tV/kg	MV	pH	PaO_**2**_	PaCO_**2**_	RR	PEEP	tV/kg	MV	pH	PaO_**2**_	PaCO_**2**_
Baseline	**25**	**5**	**6**	**5.68** ± 0.5	**7.43** ± 0.06	**245** ± 20.6	**41** ± 3.9	**25**	**5**	**6**	**5.47** ± 0.29	**7.47** ± 0.05	**248** ± 24.2	**38.9** ± 6.4
AfterVILI	**25**	**5**	**8** ± 2.1	**6.04** ± 0.41	**7.46** ± 0.15	**144** ± 61.4	**40.4** ± 18.9	**25**	**5**	**6**	**5.47** ± 0.29	**7.51** ± 0.15	**130** ± 62.5	**35.2** ± 14.1
1st hour	**34** ± 10.5	**15**	**6.5** ± 1.5	**6.57** ± 0.7	**7.38** ± 0.05	**237** ± 26.5	**46.7** ± 6	**36**	**15**	**6**	**5.47** ± 0.29	**7.38** ± 0.03	**220** ± 25.2	**49** ± 4
2nd hour	**37.2** ± 10.6	**15**	**6.8** ± 2.3	**6.62** ± 0.32	**7.39** ± 0.05	**235** ± 24.6	**47.4** ± 7.9	**36**	**15**	**6**	**5.47** ± 0.29	**7.39** ± 0.03	**227** ± 25	**49.4** ± 3.6
3rd hour	**41** ± 9.8	**15**	**6.1** ± 1.8	**6.62** ± 0.13	**7.44** ± 0.03	**245** ± 33.6	**41.3** ± 5.5	**36**	**15**	**6**	**5.47** ± 0.29	**7.39** ± 0.02	**231** ± 24.7	**49** ± 3.9
4th hour	**41** ± 8.6	**15**	**6.4** ± 2.5	**6.65** ± 0.75	**7.44** ± 0.02	**245** ± 25.7	**42.4** ± 3	**36**	**15**	**6**	**5.47** ± 0.29	**7.4** ± 0.02	**233** ± 21.8	**48.5** ± 3.6
5th hour	**41** ± 7.5	**15**	**6.9** ± 3.1	**6.82** ± 0.41	**7.47** ± 0.05	252* ± 18.5	**39.4** ± 6.4	**36**	**15**	**6**	**5.47** ± 0.29	**7.39** ± 0.02	**225** ± 12	**49** ± 3.7
6th hour	**41.8** ± 6.2	**15**	**6.2** ± 0.3	**6.74** ± 0.55	**7.47** ± 0.06	253* ± 17	**38.9** ± 6.2	**36**	**15**	**6**	**5.47** ± 0.29	**7.41** ± 0.02	**233** ± 19.7	**46.7** ± 3.1

Measure units: RR (respiratory rate) bpm, tV/kg (tidal volume/kilogram) ml/kg, MV (minute ventilation) L/min, PaO_2_ mmHg, PaCO_2_ mmHg

* comparison between SB and MV group; *p* < 0.05 (indicated in the cells of both groups)

### Hemodynamics and respiratory variables

The global hemodynamic profile was similar in the two study groups and remained stable throughout the experiment (Table 2).

**Table 2:** Hemodynamics parameters reported as Mean ± Standard Deviation in both groups

	SB group									MV group								
	MAP	mPAP	PAOP	CO	Heart rate	CVP	IAP	Arterial Lactate	EtCO_**2**_	MAP	mPAP	PAOP	CO	Heart rate	CVP	IAP	Artrial Lactate	EtCO_**2**_
Baseline	**80.3** ± 6.7	**15.2** ± 1.6	**8.5** ± 3.1	**2.6** ± 0.6	**88** ± 18	**5.5** ± 1.9	**9.7** ± 5.4	**1.7** ± 0.9	**41.5** ± 4.4	**81.7** ± 13.2	**14.9** ± 1.7	**8** ± 1.4	**2.3** ± 0.6	**82** ± 17	**5.1** ± 1.1	**6** ± 2.7	**1.3** ± 0.4	**38.1** ± 5.8
AfterVILI	**78.5** ± 7.1	**18.3** ± 4.2	**10.7** ± 5	**2.8** ± 0.7	**88** ± 16	**6.3** ± 2.1	**8.7** ± 2.5	**2.25** ± 0.9	**20.4** ± 4.7	**76** ± 10.9	**17.4** ± 5.1	**6.9** ± 1.5	**2.9** ± 0.8	**97** ± 12	**5.4** ± 1.4	**6.4** ± 1.8	**2** ± 0.7	**21.1** ± 5.9
1st hour	**77** ± 8.3	**20.5** ± 1.9	12 ** ± 3.6	**2.7** ± 0.6	83 ** ± 6	**8.3** ± 2.1	**10.5** ± 7	**1.5** ± 0.7	**45.2** ± 10.2	**80.6** ± 9.5	**20.7** ± 2.1	9.6 ** ± 0.8	**2.5** ± 0.5	99 ** ± 16	**7.3** ± 1.1	**6.4** ± 2.1	**1.4** ± 0.4	**45.4** ± 3.5
2nd hour	**77.5** ± 7.3	**20.5** ± 3.2	11 ** ± 1.8	**2.9** ± 0.9	92 ** ± 6	**8.8** ± 2.1	**9** ± 4	**1.3** ± 0.7	**46.5** ± 11.1	**79.3** ± 10.4	**21** ± 1.6	9.4 ** ± 1.1	**2.3** ± 0.4	101** ± 18	**8.1** ± 1.6	**6.4** ± 2	**1.1** ± 0.3	**46.9** ± 4.1
3rd hour	**80.2** ± 6.9	**21.5** ± 1.5	11.2** ± 2.6	**2.9** ± 0.8	94 ** ± 11	**8.3** ± 2.9	**10.7** ± 4.8	**1.3** ± 0.7	39.7 * ± 5.7	**79.4** ± 10.9	**21** ± 2.2	9.7 ** ± 0.5	**2.8** ± 0.6	104** ± 14	**7.4** ± 1	**5.7** ± 1.1	**1.1** ± 0.2	47 * ± 3.7
4th hour	**81.8** ± 8.4	**21** ± 1.8	12.8 * ± 2.6	**2.6** ± 0.3	92 ** ± 7	**8.7** ± 3.1	**10** ± 4.2	**1** ± 0.1	40 ** ± 7.1	**83.1** ± 14.4	**20.5** ± 2.4	9.7 * ± 0.5	**2.7** ± 0.6	107** ± 14	**7.6** ± 0.8	**5.8** ± 2.1	**1.1** ± 0.3	46.3** ± 3.9
5th hour	**82.5** ± 12.4	**21.2** ± 1	12.5 * ± 2.1	**2.7** ± 0.6	91 ** ± 8	**8.8** ± 2.9	**9.5** ± 5.4	**1.2** ± 0.5	40.5** ± 6.7	**80.1** ± 11.8	**20.5** ± 1.9	9.6 * ± 1	**2.7** ± 0.4	100** ± 13	**7.9** ± 0.9	**6.9** ± 2.4	**1** ± 0.3	46.7** ± 5.4
6th hour	**85** ± 8.2	**21.2** ± 1.2	13 * ± 1.4	**2.6** ± 0.2	88 * ± 10	**9.3** ± 3	**9.5** ± 4.7	**1.2** ± 0.5	43 ** ± 6.5	**78.3** ± 10.2	**20.6** ± 2.8	9.8 * ± 1	**2.6** ± 0.6	101 * ± 15	**8** ± 1.2	**5.7** ± 0.8	**1** ± 0.3	45.6** ± 5.3

Measure units: MAP (mean arterial pressure) mmHg, mPAP (mean pulmonary artery pressure) mmHg, PAOP (pulmonary artery occlusion pressure) mmHg, CO (cardiac output) L/min, Heart rate bpm, CVP (central venous pressure) mmHg, IAP (intra-abdominal pressure) mmHg, Arterial Lactate mmol/L, EtCO_2_ mmHg

* comparison between SB and MV group; *p* < 0.05 (indicated in the cells of both groups)

** comparison between SB and MV group; *p* < 0.1 (indicated in the cells of both groups)

Mean pulmonary arterial pressure, heart rate and central venous pressure showed an increase over time, but without any difference between the groups.

IAP was higher in the SB group at baseline and maintained a similar trend throughout the experiment, although the difference was not statistically significant.

The value of PAOP was similar in the study groups at baseline. It increased in both groups (time factor: *p* < 0.001) but was higher in the SB group during the experiment (*p* = 0.018). The gap between the MV and SB groups enhanced over time, becoming more evident during the last 3 h of observation.

No difference in fluid balance between the groups was underlined.

EtCO_2_ had different trends in the two groups (time*group effect *p* = 0.04); the level was higher in the MV group at 3 h (*p* = 0.02) but the difference between groups decreased thereafter.

### Inflammation

**Fig. 1:**
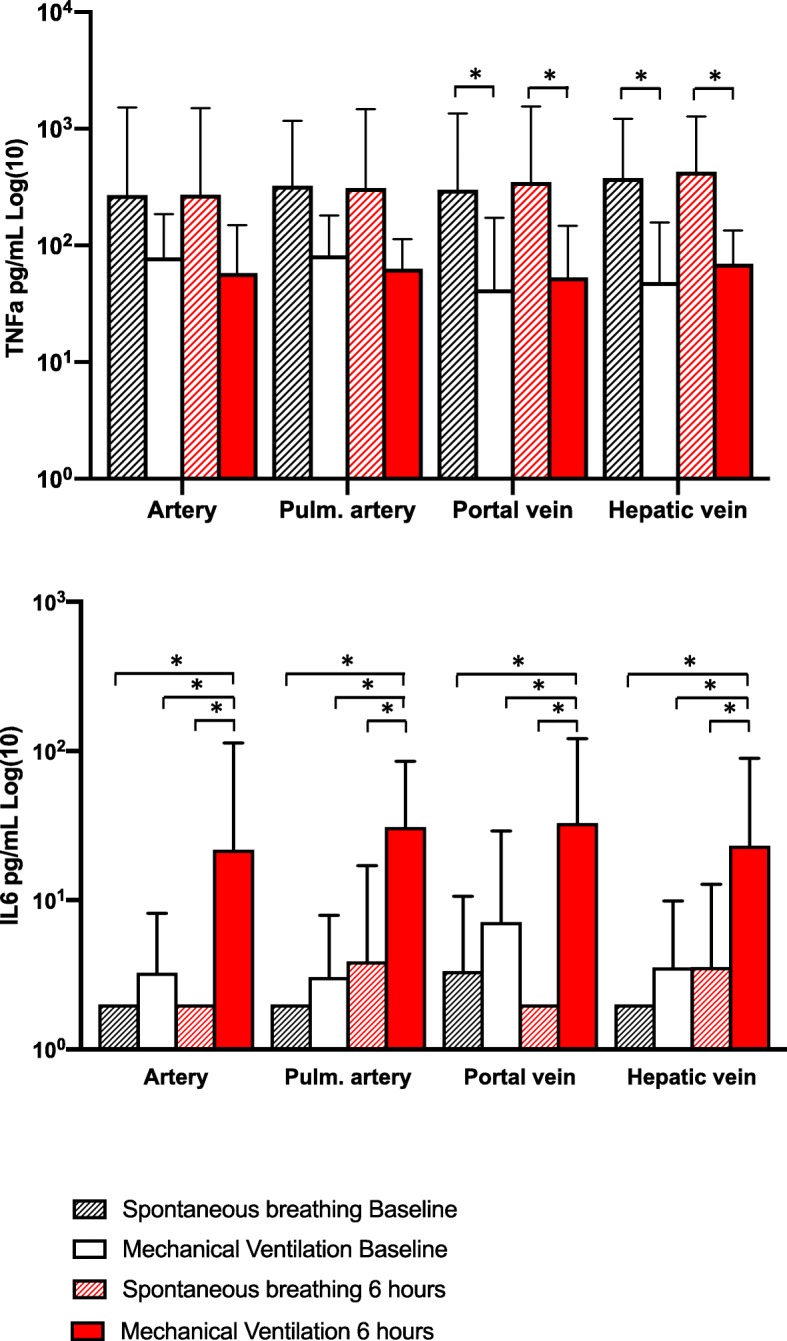
Cytokine concentrations (log-transformed) in the study groups at baseline and 6 h after ARDS induction

No difference was found between the MV and SB groups in IL6 at baseline, but the IL6 concentration at 6 h was higher in the MV group (Fig. 1) in all vascular beds.

TNFα was higher in the SB group compared with the MV group in the portal and hepatic veins at baseline and after 6 h of observation (Fig. 1). Similar trends were seen in all vascular beds.

IL1b was below the detection threshold in all the samples.

**Fig. 2:**
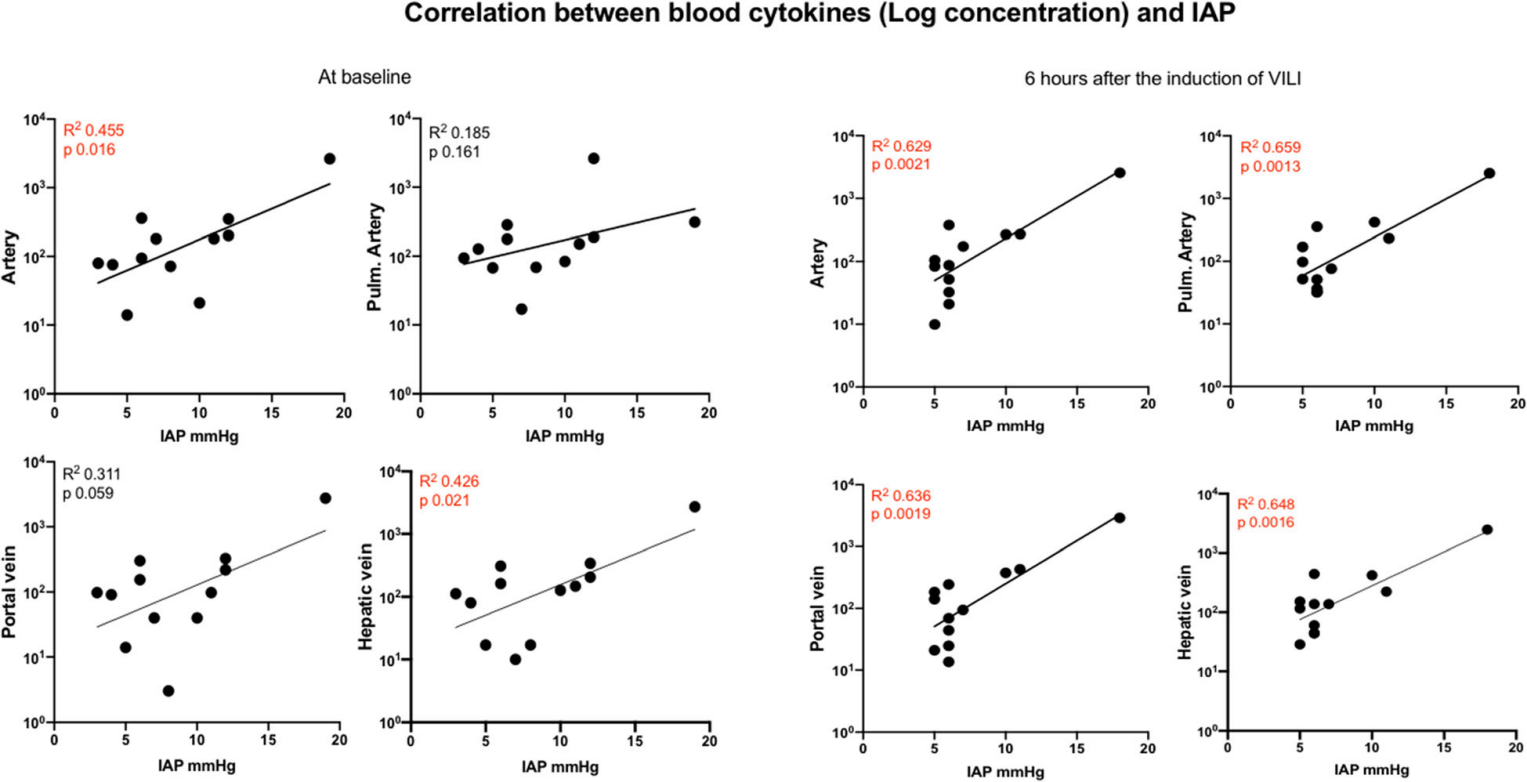
Correlation between TNFα (Log10) and IAP mmHg in both groups at baseline and 6 h after ARDS induction

TNFα in blood correlated to IAP at 6th hour of observation (Fig. 2).

The increase of IL6 from pulmonary artery to systemic artery blood at 6 h was of 190% ± 45 for the SB and 140% ± 30 for the MV group, with no difference between the groups. In addition, IL6 increased of 12% passing through the liver in both groups (SD was 26 in the SB group and 38 in the MV group).

The difference of TNFα concentration from pulmonary artery to systemic blood was lower (*p* = 0.05) in the SB group: - 17% ± 3 (TNFα concentration decreased from the pulmonary artery to the arterial blood); MV group: + 26% ± 3 (TNFα concentration increased).

The TNFα concentration decreased from portal vein to hepatic vein at 6 h after ARDS induction without any difference between groups (− 48% ± 10 for the SB group and – 61% ± 13 for the MV group)*.*

**Fig. 3:**
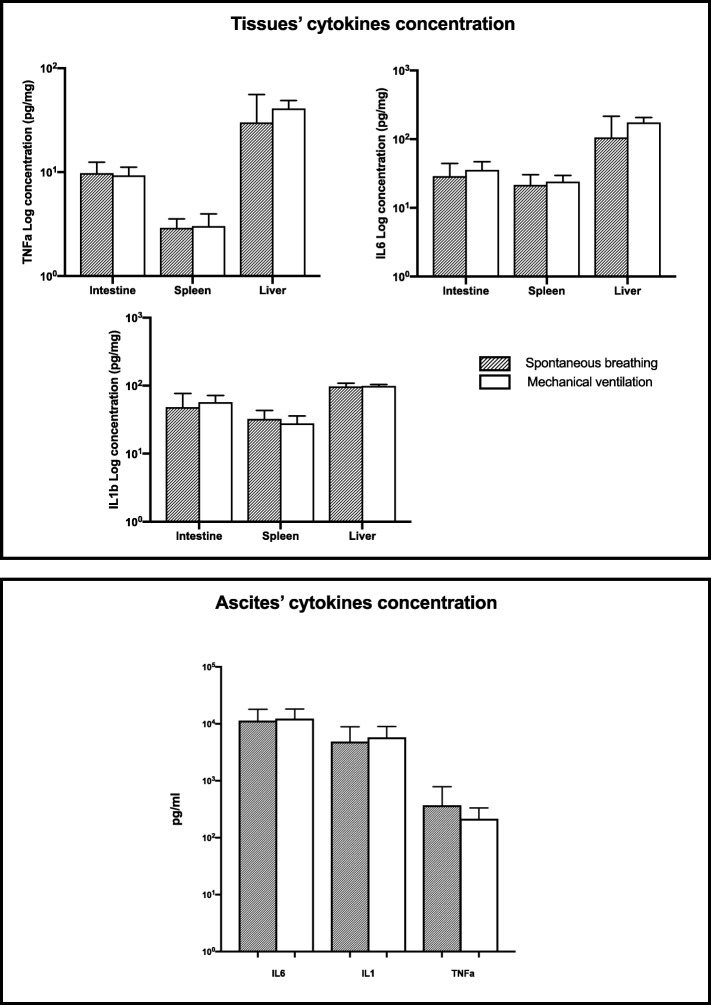
Cytokine concentration in ascites (pg/ml) and tissues (duodenum, spleen and liver)

Cytokine concentration in tissues (duodenum, spleen, liver) did not show any difference between the two groups (Fig. 3).

No difference was detected in the two groups for ascites for any of the cytokines measured (Fig. 3).

Inflammation of abdominal organs was assessed using an inflammatory score on histopathological samples of abdominal organs/tissue and measurement of cytokines.

The inflammatory score did not show any difference between groups in any of the abdominal organs studied.

### Edema

**Fig. 4:**
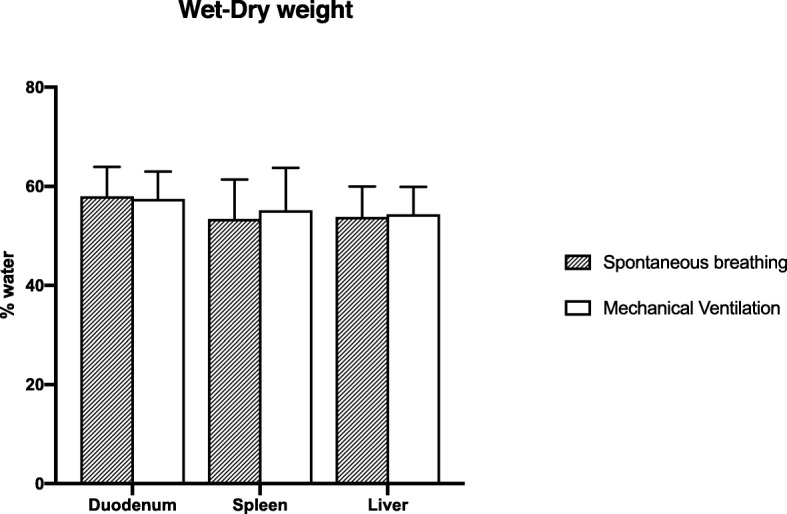
Edema in the tissues of abdominal organs (wet-dry weight). The results are reported as % of water weight over the total weight of the sample

No difference (by either histopathological estimation or in wet-dry weight) in edema formation in the abdominal organs was detected between the two groups (Fig. 4).

Hemoglobin concentration over time was similar in the MV and SB groups.

## Discussion

In this model of experimental ARDS, MV does not increase edema or inflammation in the abdominal compartment when compared with SB. However, with the settings applied in this study MV induced pulmonary inflammation as indicated by IL6 and TNFα responses.

A double-hit ARDS model consisting of lung lavage followed by injurious ventilation was used. Such a model, which has been used extensively, mirrors many of the characteristics found in early human ARDS [20, 21]. The severity of the lung injury was restricted to mild to moderate to prevent the animals from failed SB. The inflammatory reaction localizes primarily to the lungs and produces no initial systemic symptoms. The ventilation was kept the same in both groups with no difference in PEEP, RR or V_T_s. PEEP was set to a high level in both groups to facilitate SB in the animals.

The hemodynamics was similar in the two groups, except that pulmonary artery occlusion pressure, despite equal fluid balance, tended to be higher in the SB group.

Based on previous findings, we expected to find that MV would trigger abdominal inflammation, even when there was no primary systemic inflammation. However, it was not possible to demonstrate any increased inflammation or edema in the abdominal organs. Moreover, increased capillary leakage was not found, as estimated by hemoglobin concentration and wet-dry weight. On the other hand, elevated serum IL6 and TNFα in the MV group indicate that MV, despite protective settings, induced production of inflammatory mediators in the lungs. The pulmonary inflammatory reaction did not propagate to the abdominal organs.

In contrast, in inflammatory primed abdominal organs, as was the case in previous studies, the inflammatory mediators produced by MV from the lungs probably worked as a “second hit” and amplified the already existing abdominal edema and inflammatory reaction. Indeed, this contention mirrors clinical reality. Because ARDS is clinically caused by an underlying inflammatory condition (e.g., sepsis, pneumonia, trauma or pancreatitis), the augmentation of the pulmonary inflammation by VILI could disseminate systemically and enhance inflammation in compromised abdominal organs (such as the liver or the bowel) causing multiple organ failure [22, 23]. Inarguably, multiple organ failure is the most common cause of death in ARDS [24].

Another difference from earlier studies is that PEEP is set at the same high level in both groups. A lower PEEP would theoretically improve the lymphatic drainage through thoracic duct and reduce the splanchnic venous stasis and thus decrease edema formation. In a previous study PEEP in a SB group was set a 5 cmH_2_O and edema formation was significantly higher with MV than with SB, even when the groups had the same low PEEP level. PEEP was found to worsen edema further. That no difference was found between the SB and MV groups with a high PEEP level may indicate that high PEEP impairs edema clearance in the same way, no matter the ventilation mode. Moreover, in several studies RR was higher with SB, and it is known that diaphragm movement creates a pump effect on the thoracic duct [25]. Thus, the increased RR in the SB group could have been the result of an edema-diminishing/lymph drainage-enhancing factor.

The better cytokine profile in the SB group in this study is also reflected in the improvement in gas exchange in which both PaO_2_ and PaCO_2_ improved faster in the SB group, indicating that SB reduced lung collapse and increased lung compliance, as demonstrated in similar models in a previous studies [26]. Besides, earlier findings suggest that the high level of PEEP associated with spontaneous breathing could have contributed to the improvement of gas exchange and the reduction of lung damage [27].

## Limitation of the study

Some limitations of the study need to be addressed. First, this is an animal ARDS model with all its inherent limitations and the number of animals is limited in order to comply with the ARRIVE Guidelines, therefore the results can be generalized to patients only with extreme caution. The study aimed to examine whether “protective” MV would induce splanchnic inflammation under non-septic ARDS conditions; however, in human patients ARDS is always due to an underlying, usually severe, inflammatory condition. Second, the observation time may have been too short to detect a full response in abdominal edema and inflammation. Nevertheless, 6 h of observation should have been enough time to see a signal in the cytokine response.

Finally, we did not sample lung tissue. Still, previous studies using a similar model have shown pulmonary edema and local cytokine production [15, 28].

## Conclusions

In this porcine non-septic ARDS model MV did not augment abdominal inflammation or edema. However, protective MV promoted pulmonary inflammation as estimated by the cytokine response. These results suggest that SB, with adequately high CPAP, could be preferred in selected patients with ARDS.

## Data Availability

The datasets used and analyzed during the current study are available from the corresponding author on reasonable request.

## Abbreviations

APP: Abdominal perfusion pressure
CO: Cardiac output
CVP: Central venous pressure
DW-MRI: Diffusion weighted magnetic resonance imaging
ELISA: enzyme-linked immunosorbent assay
HR: Heart rate
IAH: Intra-abdominal hypertension
IAP: Intra-abdominal pressure
IL1b: Interleukin 1b
IL6: Interleukin 6
MAP: Mean arterial pressure
mPAP: Mean pulmonary arterial pressure
MV: Mechanical ventilation
PaCO_2_: Carbon dioxide arterial pressure
PaO_2_: Oxygen arterial pressure
PAOP: Pulmonary artery occlusion pressure
PEEP: Positive end-expiratory pressure
RR: Respiratory rate
SB: Spontaneous breathing
SvO_2_: Mixed venous oxygen saturation
TNFα: Tumor necrosis factor alpha
V_T_: Tidal volume
VILI: Ventilation induced lung injury
